# The Mectizan^® ^Donation Program – highlights from 2005

**DOI:** 10.1186/1475-2883-5-11

**Published:** 2006-09-27

**Authors:** Mary M Alleman, Nana AY Twum-Danso, Björn I Thylefors

**Affiliations:** 1 The Mectizan^® ^Donation Program, 750 Commerce Drive, Suite 400, Decatur, GA 30030, USA

## Abstract

Through the Mectizan^® ^Donation Program, Merck & Co., Inc. has donated Mectizan (ivermectin, MSD) for the treatment of onchocerciasis worldwide since 1987. Mectizan has also been donated for the elimination of lymphatic filariasis (LF) since 1998 in African countries and in Yemen where onchocerciasis and LF are co-endemic; for LF elimination programs, Mectizan is co-administered with albendazole, which is donated by GlaxoSmithKline. The Mectizan Donation Program works in collaboration with the Mectizan Expert Committee/Albendazole Coordination, its scientific advisory committee. In 2005, a total of 62,201,310 treatments of Mectizan for onchocerciasis were approved for delivery via mass treatment programs in Africa, Latin America, and Yemen. Seventy-seven percent and 20% of these treatments for onchocerciasis were for countries included in the African Programme for Onchocerciasis Control (APOC) and the former-Onchocerciasis Control Programme in West Africa (OCP), respectively. The remaining 3% of treatments approved were for the six onchocerciasis endemic countries in Latin America, where mass treatment is carried out twice-yearly with the goal of completely eliminating morbidity and eventually transmission of infection, and for Yemen. All 33 onchocerciasis endemic countries where mass treatment with Mectizan is indicated have ongoing mass treatment programs. In 2005, 42,052,583 treatments of co-administered albendazole and Mectizan were approved for national Programs to Eliminate LF (PELFs) in Africa and Yemen. There are ongoing PELFs using albendazole and Mectizan in nine African countries and Yemen; these represent 35% of the total number of countries expected to require the co-administration of these two chemotherapeutic agents for LF elimination. In Africa, the expansion of existing PELFs and the initiation of new ones have been hampered by lack of resources, technical difficulties with the mapping of LF endemicity, and the co-endemicity of LF and loiasis. Included in this review are recommendations recently put forward for the co-administration of albendazole and Mectizan in areas endemic for LF, loiasis, and onchocerciasis.

## Review

### Introduction

On the occasion of the 35^th ^Mectizan^® ^Expert Committee/Albendazole Coordination meeting (MEC/AC35) which took place in London, United Kingdom from 10–12 January 2006, a review of the work of the Mectizan Donation Program (MDP) in onchocerciasis (river blindness) control and lymphatic filariasis (LF) elimination during 2005 was presented. Highlights from that review are summarized below. The MDP is an international public health organization supported by Merck & Co., Inc. to oversee the donation of Mectizan (ivermectin, MSD) for onchocerciasis control worldwide and by Merck and GlaxoSmithKline (GSK) to oversee their donations of Mectizan and albendazole, respectively, for LF elimination in the countries where the two diseases are co-endemic.

### Background

In 1987, Merck announced the donation of Mectizan for the treatment of onchocerciasis worldwide for as long as necessary. The following year, Merck established the Mectizan Expert Committee (MEC); the MEC is an independent, advisory committee of seven recognized experts in the field of international public health and/or tropical medicine charged with providing advice and guidance in developing policies and procedures to assure the safe, effective, and appropriate use of Mectizan. Shortly thereafter, the MDP was created to serve as the secretariat for the MEC and to manage the day to day operations of the donation [1].

In 1998, Merck expanded its donation of Mectizan for use in national Programs to Eliminate LF (PELFs) in 28 African countries and in Yemen where onchocerciasis and LF are co-endemic [1]. When used in PELFs, a single dose of Mectizan is co-administered with a single dose of albendazole [2-4]. GSK has appointed two LF technical advisors to work with the MEC in making technical and programmatic decisions regarding the co-administration of Mectizan with albendazole for LF; as a consequence, the committee is now known as the Mectizan Expert Committee/Albendazole Coordination. The MEC and MEC/AC have met on a regular basis since their respective inceptions.

### Treatment indications and eligibility

Mectizan is indicated for the treatment of onchocerciasis caused by *Onchocerca volvulus *and for the treatment of the microfilaremia caused by infection with *Wuchereria bancrofti*, the causative agent of LF in Africa [5]. The oral dose of Mectizan recommended for mass treatment programs for the control of onchocerciasis or for the elimination of LF is approximately 150–200 micrograms (μg)/kilogram (kg) of bodyweight [3,4]. Each tablet of Mectizan contains 3 milligrams (mg) of ivermectin, and such tablets are referred to as "3 mg tablets" [5]. The number of 3 mg tablets needed to achieve the recommended dose is determined by an individual's bodyweight or height [5]. If bodyweight is used as the dosing criteria, those weighing less than 15 kg are ineligible for treatment; while if height is used, those less than 90 centimeters tall are ineligible. Others ineligible for treatment with Mectizan are pregnant women, women breast-feeding infants less than one week old, individuals with serious illnesses of an acute or chronic nature, and individuals with a history of hypersensitivity response to Mectizan [4]. Using annual treatment and Mectizan usage data from mass treatment programs over the years, the MDP has calculated that the typical number of 3 mg tablets used per person treated is approximately three (average dose).

When used in PELFs, the recommended dose of Mectizan (150–200 μg/kg bodyweight) is provided with a standard dose of albendazole (one 400 mg tablet per person), and the same ineligibility criteria used for Mectizan are applied [3,4].

In this article, the expressions "approved treatments" and "treatments approved" (and variations thereof) for onchocerciasis correspond to the number of average doses of Mectizan approved in a stated year and do not correspond to the number of individuals to be treated since, in several programs, individuals are treated more than one time per year. When these expressions are used with regard to PELFs, they correspond to the number of average doses of Mectizan and standard doses of albendazole approved in a stated year and do correspond to the number of individuals to be treated since individuals are treated only one time per year in PELFs.

### Onchocerciasis overview

**Figure 1:**
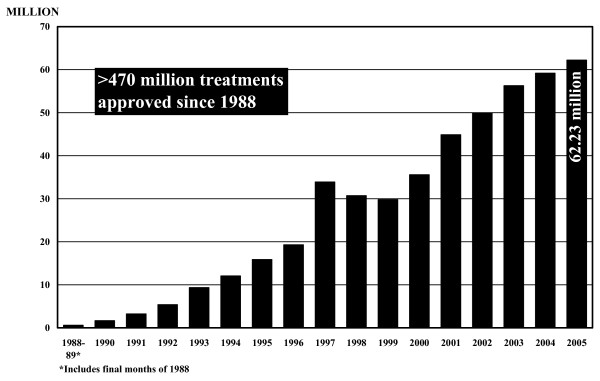
**Treatments with Mectizan Approved for Onchocerciasis, 1988–2005 (Humanitarian Donation and Mass Treatment Programs Combined)**. The figure in the 2005 column refers to the total number of treatments with Mectizan approved (rounded to the nearest 10,000) during the 2005 calendar year for onchocerciasis for mass treatment programs and humanitarian donations combined. For any given year indicated in this figure, the year of treatment approval may be different from the year during which treatments actually occurred.

In 2005, the MEC approved 62,201,310 treatments with Mectizan for onchocerciasis to be delivered via mass treatment programs in endemic countries. An additional 31,200 treatments were approved via the Humanitarian Donation Program (HDP) which serves the needs of physicians, clinics, and small organizations around the world in need of Mectizan for the treatment of individuals with onchocerciasis in areas where mass treatment is not being implemented; these account for 0.05% of the overall treatments approved (62.23 million) for onchocerciasis in 2005 (Figure 1).

**Figure 2:**
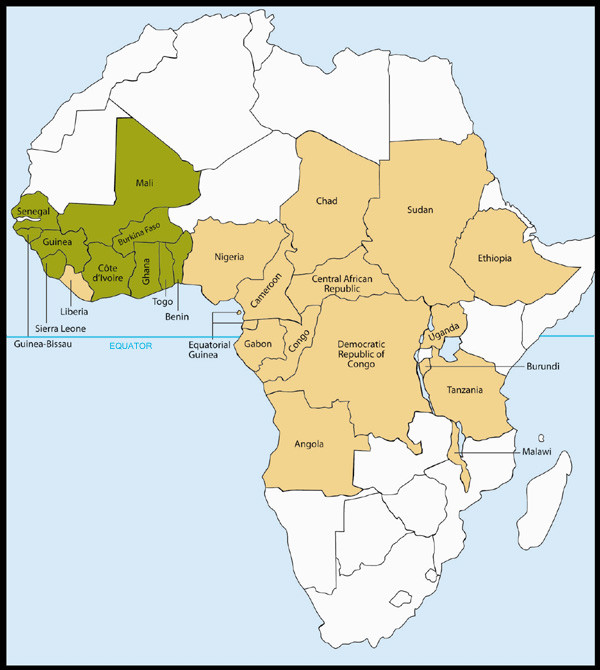
**The 26 Countries in Africa where Mass Treatment with Mectizan is Indicated and Ongoing for Onchocerciasis, as of the end of 2005**. Countries are colored coded according to their inclusion in the former-OCP () or APOC () regions. As of the end of 2005, there were mass treatment programs with Mectizan for onchocerciasis in all 26 African countries where such intervention is epidemiologically justified. These 26 countries, plus Niger and Mozambique, are eligible for Mectizan combined with albendazole for national PELFs. This map is reproduced with permission of the *Annals of Tropical Medicine and Parasitology*, 2006, Volume 100, pages 733–46.

**Figure 3:**
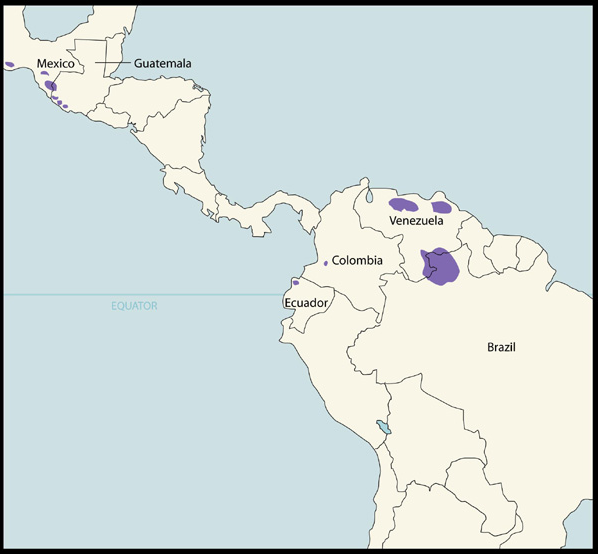
**Foci in Latin America (OEPA region) where Onchocerciasis is Endemic and where Mass Treatment with Mectizan is Indicated and Ongoing, as of the end of 2005**. In Latin America (OEPA region), as of the end of 2005, all onchocerciasis endemic foci were indicated for twice-yearly mass treatment with Mectizan, regardless of endemicity, as a strategy for the elimination of onchocercal morbidity and transmission of infection. This map is reproduced with permission of the *Annals of Tropical Medicine and Parasitology*, 2006, Volume 100, pages 733–46.

Since 1988, when Merck made its first donations, enough Mectizan has been provided for over 467 million treatments for onchocerciasis for delivery via mass treatment programs; if these treatments are combined with those approved cumulatively through the HDP, the overall total is over 470 million as of the end of 2005 (Figure 1). Mass treatment with Mectizan for onchocerciasis is currently ongoing in each of the 33 endemic countries where this treatment strategy is justified by the particular epidemiological situation (i.e. ≥ 40% positive skin snip prevalence, ≥ 20% onchocercal nodule prevalence in adult males ≥ 20 years of age, maintenance of previous control efforts, or as a strategy for the interruption of transmission) [6-10]. Figures 2 and 3 illustrate the 26 countries in Africa and the foci in the six endemic Latin American countries, respectively, where mass treatment with Mectizan for onchocerciasis is currently indicated and ongoing; the African countries are color-coded to reflect their inclusion in the former Onchocerciasis Control Programme in West Africa (OCP) or African Programme for Onchocerciasis Control (APOC) regions. The 33^rd ^country with ongoing mass treatment with Mectizan for onchocerciasis is Yemen which is not depicted in Figure 2 or 3.

### Africa

#### APOC region

APOC is a regional program established in 1995 and executed by the World Health Organization (WHO) with the support of the World Bank and the private and public sectors. Before 2010 when its operations are scheduled to close, APOC will strive to support the establishment of effective and self-sustainable, annual, community based mass treatment with Mectizan in all onchocerciasis endemic foci in countries in Africa in need of such intervention and that were not included in the OCP [11,12]. APOC's ultimate goal is 'to eliminate onchocerciasis as a disease of public-health importance and an important constraint to socio-economic development throughout Africa' [11].

**Figure 4:**
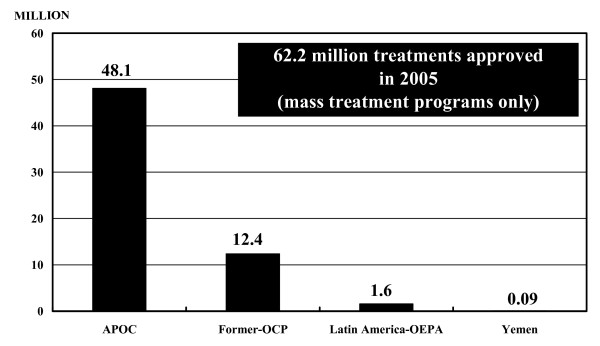
**Treatments with Mectizan Approved for Onchocerciasis (Mass Treatment Programs Only) by Region, 2005**. The figure on top of each bar refers to the number of treatments (in millions) with Mectizan approved for delivery via mass treatment programs for onchocerciasis for the particular region or country indicated. All treatment approval figures have been rounded to the nearest 100,000, with the exception of that for Yemen (rounded to the nearest 10,000). The year of treatment approval may be different from the year during which treatments actually occurred.

The greatest proportion of treatments with Mectizan approved by the MEC for delivery via mass treatment programs for onchocerciasis in 2005 (77.4%) was for countries in the APOC region (Figures 2 and 4). Among the approvals in the region were treatments with Mectizan for a new program in the Extreme North Province, Cameroon and for the re-launching of two programs, in the Bururi and Rutana foci in Burundi, each having had a period of interruption due to civil unrest.

#### Former-OCP region

The countries in the former-OCP region began incorporating treatment with Mectizan into OCP activities, namely vector control, shortly after the announcement of the donation was made [7,13]. The drug was first distributed where onchocercal blindness was most prevalent in the program area [7,13]. Its use was later expanded to complement the program's ongoing larviciding activities and into program areas where larviciding was not epidemiologically justified or cost-effective but control measures were indicated [7,13]. In 1991, eleven years prior to the formal closure of the OCP, its Devolution Unit began the process of transferring the full responsibility for onchocerciasis control and surveillance activities to the ministries of health of each of the 11 member countries where that responsibility remains today [13].

Mass distribution of Mectizan for onchocerciasis is currently indicated in foci in all of the former-OCP countries, with the exception of Niger, for the purpose of maintaining the OCP's accomplishments (Figure 2) [7-9,13,14]. Moreover, in recent years, mass treatment with Mectizan for onchocerciasis has been extended into forested regions of some former-OCP countries; such regions were not included in the OCP's operations because blinding onchocerciasis (OCP's priority) was less prevalent. In Niger, where the numbers of individuals at risk for onchocerciasis are too few to justify mass treatment, clinic based treatment is provided for those infected. Treatment with Mectizan was approved in 2005 for the re-launching of mass treatment of onchocerciasis in Guinea-Bissau which will be integrated with the country's newly established, national PELF.

Intensified control efforts have been instituted in the "Special Intervention Zones" (SIZ) where, for various reasons, there were unsatisfactory program results at the end of 2002 when the OCP formally closed [9]. The SIZ include the basins of the Pru river in Ghana, the tributaries of the Oti and Upper Oueme rivers in Togo/Benin, the Mafou/Upper Niger and Tinkisso river basins in Guinea-Conakry, and the entirety of Sierra Leone; the latter being included due to the interruption of control activities during the period of civil unrest in the 1990s and early 2000s [9]. The goal of operations in the SIZ is to bring epidemiological and entomological parameters to an acceptable level [9]. Annual Mectizan treatment with high coverage (85% of the total population) is recommended for all eligible communities within the SIZ, with the communities in the Oti, Mafou, and Tinkisso basins receiving a second round of treatment each year and aerial larviciding occurring in the Oti and Oueme river basins [7,9,15]. In addition, twice-yearly treatment with Mectizan also occurs in selected onchocerciasis endemic foci in Burkina Faso and Senegal [7].

Twenty percent of the treatments with Mectizan approved by the MEC for delivery via mass treatment programs for onchocerciasis in 2005 were for countries in the former-OCP region; this is sufficient Mectizan for 12.4 million treatments (Figures 2 and 4). Mass treatment with Mectizan at the same magnitude is projected for this region for the foreseeable future or at least until there is evidence that treatment can cease without the risk of disease recrudescence [8,9,13,14]. A United Nations Development Program/World Bank/WHO Special Programme for Research and Training in Tropical Diseases (TDR) sponsored study is underway in five foci in the former-OCP region where the prevalence of onchocerciasis is currently very low to determine if local elimination of transmission has occurred and if, consequently, mass treatment with Mectizan can be stopped [16].

### Latin America-OEPA region

The Onchocerciasis Elimination Program for the Americas (OEPA) is a multi-national and multi-agency coalition established in 1993 that has the goal of completely eliminating ocular morbidity attributable to onchocerciasis and transmission of infection in the 13 endemic foci found in six countries in Latin America (Figure 3) [10,17]. It is estimated that just over 500,000 people are at risk of infection with *O. volvulus *in the region [18]. OEPA's strategy is the twice-yearly treatment with Mectizan of individuals in all onchocerciasis endemic communities in the 13 foci, regardless of the community's endemicity. As part of the strategy, in each treatment round, national programs strive to treat at least 85% of the population eligible for treatment with Mectizan in each endemic focus as that should lead to interruption of transmission in the Latin American setting [10,18].

As of 2005, the population eligible for treatment with Mectizan for onchocerciasis in the region's 13 foci combined was approximately 454,000 people [10]. For the last three years (2003–2005), in all foci in the region, with the exception of the southern focus of Venezuela, greater than 85% of the eligible population have been treated [10]. In 2005, the MEC approved 1,547,006 treatments with Mectizan for onchocerciasis for the region, 2.5% of the total treatments approved for delivery via mass treatment programs for the year (Figure 4). For logistical reasons, in 2005 national programs in the region were offered the option of receiving sufficient Mectizan for two years of treatment, rather than the standard practice of annual supply; thus, the total treatments approved for the year exceed the region's annual treatment objective of approximately one million treatments (total eligible population X 2 cycles of treatment).

Epidemiological and entomological data on *O. volvulus *and *Simulium *species and opthalmological examinations in the region indicate that in selected foci in Colombia, Ecuador, Guatemala, and Mexico transmission of infection may have already been interrupted [10,18]. The possibility that transmission has already been interrupted in several foci and the virtual elimination of new cases of blindness due to onchocerciasis in the region have led OEPA to contemplate: i) the possible focal cessation of mass treatment with Mectizan, ii) the process for documentation of the elimination of *O. volvulus *transmission, and iii) the post-treatment surveillance that will be needed [10, personal communication M. Sauerbrey]. The region is currently reviewing the criteria for the certification of interruption of transmission of human onchocerciasis put forth by WHO, after a meeting of experts in 2001, to determine how they should be applied to the Latin American setting [19].

### Yemen

Onchocerciasis in Yemen has been described as occurring in villages near main wadis (i.e. seasonal watercourses) with permanent, westward flowing streams and some associated tributaries, at altitudes between 300 and 1200 meters [20]. Available data indicate that the southern and northern limits of onchocerciasis distribution are Wadi Ghail in the southwest and Wadi Surdud in the northwest, respectively [20]. Approximately 30,000 people are estimated to be infected with *O. volvulus *in the country [21]. A particular dermal manifestation of onchocerciasis, known as sowda, is commonly observed in Yemen. In those with sowda, a single limb (often a leg) is typically affected with severe pruritis, moderate edema, pachydermia, papular or pustular eruption, and darkening of skin color [20]. In addition, enlargement of femoral lymph nodes can be seen [20]. Mectizan has been recommended for use at 3-monthly intervals to control the clinical symptoms of sowda, and this strategy has since been adopted in Yemen [22]. The distribution of Mectizan for onchocerciasis in Yemen is primarily passive whereby those who want treatment to relieve symptoms present to drug distributors. In areas where onchocerciasis is co-endemic with LF, one annual cycle of co-administered Mectizan and albendazole is provided through Yemen's national PELF whereby all eligible community members in the program area are included in treatment regardless of their individual onchocerciasis or LF infection status; the additional quarterly treatments with Mectizan for sowda in these co-endemic areas are provided passively to patients who seek them. In 2005, the MEC approved 91,000 total treatments with Mectizan, to be provided on a quarterly basis, for onchocerciasis in Yemen; this represents 0.2% of the total treatments approved for the year for onchocerciasis for delivery via mass treatment programs (Figure 4).

### Humanitarian Donation Program

The HDP provides Mectizan to health care professionals treating relatively small numbers of confirmed cases of onchocerciasis in hospitals and clinics worldwide and has been in existence since the donation was announced. In the first years of the donation, the HDP was an important mechanism through which Mectizan could be obtained for patients with onchocerciasis; now, more than 99% of the Mectizan needed is provided through national onchocerciasis control programs (NOCPs) where the vast majority of it is delivered via community based mass distribution and the rest via clinic based treatment. In 2005, as much as possible, requests made to the HDP from endemic countries with established NOCPs were forwarded to national coordinators for review and supply; in this way, all onchocerciasis control activities within an endemic country can be coordinated at a central level. The largest single donation made by the HDP in 2005 was Mectizan for onchocerciasis for 10,000 displaced Sudanese living in refugee camps in Kenya, a country where onchocerciasis is no longer endemic [23]. Other, smaller donations were made for the treatment of individuals with onchocerciasis in Central America, Europe, and the Middle East.

### Lymphatic Filariasis overview

Following a resolution by the World Health Assembly in 1997 to eliminate LF as a public health problem, the Global Programme to Eliminate Lymphatic Filariasis (GPELF) was launched by the WHO [2]. The recommended mass drug administration (MDA) strategy for PELFs in countries co-endemic for onchocerciasis is a standard dose of Mectizan (150–200 μg/kg bodyweight) co-administered with a standard dose of albendazole (one 400 mg tablet per person) on an annual basis for four to six years [3]; this strategy is applicable to 28 countries in sub-Saharan Africa and to Yemen (Figure 2). Diethylcarbamazine (DEC), used in PELFs in other countries with or without albendazole, is not recommended for the above-mentioned 29 countries since it can induce serious adverse events (SAEs) in individuals infected with *O. volvulus *and/or *Loa loa *[3,24].

An added complication in LF elimination strategies for Africa is the safety of the recommended chemotherapeutic drugs in loiasis-endemic areas [25-27]. Clinical and epidemiological data from Cameroon have demonstrated that individuals with *L. loa *microfilaremia of > 30,000 microfilariae/milliliter blood are at risk, albeit rarely, of developing neurological SAEs after treatment with Mectizan [28,29]. A positive linear relationship has been reported between the average intensity and the prevalence of *L. loa *infection in communities; thus community-level assessments which estimate the prevalence of infection with *L. loa *can be used to identify geographic areas where *L. loa*-associated SAEs following treatment with Mectizan are more likely to occur [30]. A spatial model of *L. loa *prevalence across west and central Africa has been developed and linked to a geographical information system to create a map that has been useful in identifying large geographic areas where the risk of SAEs could be high [31]. The map is instrumental for locating areas appropriate for further evaluation with the Rapid Assessment Procedure for Loiasis (RAPLOA), a rapid assessment tool for the presence and intensity of *L. loa *infection at the community level [32]. In *L. loa *endemic areas, mass treatment with Mectizan for onchocerciasis is carried out where the benefit of treatment is deemed to outweigh the risk of *L. loa*-associated SAEs (i.e. where the prevalence of onchocerciasis is ≥ 20% as measured by palpable onchocercal nodules or ≥ 40% as measured by skin snips) [33,34].

Where mass treatment for onchocerciasis occurs in *L. loa *endemic areas, it is implemented following the MEC/Technical Consultative Committee (TCC) guidelines for the treatment of onchocerciasis with Mectizan in areas co-endemic for loiasis [34]. The TCC is APOC's scientific advisory committee. In brief, the guidelines call for enhanced education of community members, specialized training for community volunteers and health personnel in enhanced post-treatment surveillance, referral, prompt and appropriate clinical management, and provision of designated referral centers with appropriate medical supplies in order to reduce the risk of neurological complications and death. Moreover, there are *L. loa *Technical Advisors posted in the countries where the most *L. loa*-associated SAEs have been reported; these advisors are financially supported by MDP and APOC and provide *L. loa*-related technical and clinical advice and assistance to their respective NOCPs.

There have been no data published to date indicating that Mectizan alone, or in combination with albendazole, provides a clinical benefit to individuals with *W. bancrofti *infection. Thus at the present time, the use of these drugs, in combination, in national PELFs in Africa and Yemen is for the elimination of LF transmission through successive reductions in microfilaremia with each annual treatment round [3,24,35-37]. With this strategy, the benefit accrues over time to the community but not immediately to an infected individual. As a consequence, there has been a reluctance to expand PELFs into areas co-endemic for LF and loiasis where there would be a risk of *L. loa*-associated SAEs and no documented clinical benefit to individuals with LF [25-27]. In contrast, in *L. loa *endemic areas where mass treatment with Mectizan for onchocerciasis is indicated and thus the benefit of treatment outweighs the risk of SAEs, the concern with initiating PELFs has been the addition of albendazole into ongoing mass treatment with Mectizan [38].

### MDA for lymphatic filariasis in non-loiasis endemic areas

**Figure 5:**
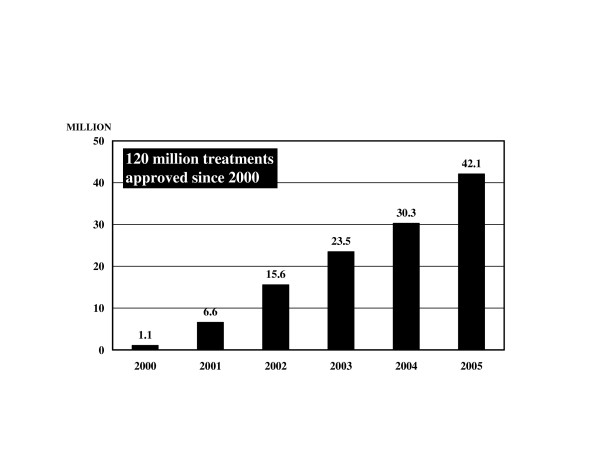
**Treatments with Albendazole and Mectizan Approved for Programs to Eliminate Lymphatic Filariasis (PELFs), 2000–2005**. The figure on the top of each bar refers to the number of treatments (in millions) with albendazole and Mectizan approved for national PELFs in Africa and Yemen in the calendar year indicated. All annual treatment approval figures have been rounded to the nearest 100,000. For any given year indicated in this figure, the year of treatment approval may be different from the year during which treatments actually occurred.

Between 2000 when the PELFs in Africa began and 2005, approximately 120 million treatments of co-administered albendazole and Mectizan were approved by the MEC/AC for nine African countries and Yemen for MDA for LF elimination (Figure 5). The nine African countries are Benin, Burkina Faso, Ghana, Guinea-Bissau, Mali, Nigeria, Tanzania, Togo, and Uganda. These 10 countries with active PELFs represent 35% of the total number of countries expected to require the co-administration of albendazole and Mectizan for LF elimination. Of note, both Benin and Nigeria have areas with *L. loa *endemicity; the treatments approved by the MEC/AC for LF elimination in these countries have excluded such areas.

In 2005, 42,052,583 treatments of co-administered albendazole and Mectizan were approved for PELFs in Africa and Yemen; this figure is 13% of the ultimate annual treatment goal projected (approximately 320 million annual treatments) when all countries eligible to receive these two drugs for PELFs in Africa and Yemen are combined (Figures 2 and 5). In Africa, the expansion of existing PELFs and the initiation of new ones have been slower than anticipated primarily due to scarce human and financial resources for field operations, technical difficulties with the tools for endemicity mapping, and the co-endemicity of loiasis and LF in many LF endemic countries [26,27,39]. As of the end of 2005, only two countries, Togo and Yemen, had scaled up MDA sufficiently to reach 100% geographic coverage, and this occurred after at least three rounds of treatment. Since the recommendation for the duration of PELF MDA is four to six years, a slow rate of scaling up translates into national PELFs of longer duration and possibly loss of political and societal momentum for the program in addition to a decrease in overall efficiency [3,27].

### MDA for lymphatic filariasis in loiasis-endemic areas

As described above, there has been concern about launching PELFs in African countries known to be, or suspected of being, endemic for *L. loa *[25-27]. However, progress has recently been made in clarifying the safety issues of conducting LF MDAs in loiasis-endemic areas. In 2005, an informal consultation of experts, sponsored by WHO/TDR, determined that there is neither a biological rationale nor available data to suggest that the addition of albendazole to Mectizan would increase the number or severity of adverse reactions if the two drugs were to be used together to treat populations co-endemic for onchocerciasis, LF, and loiasis [38]. In addition, the experts concluded that the most effective approach to addressing the safety of this drug combination in areas with triple endemicity is to use the drugs on a large scale with intensified pharmacovigilance. One of the most significant developments for LF elimination in Africa was the recent endorsement, by all of MDP's partners during MEC/AC35, of the conclusions made during the informal consultation.

All partners agreed that in view of the well recognized inverse relationship between the number of cycles of Mectizan treatment and the incidence of SAEs seen in populations with any of these filarial infections, the specifics of the enhanced pharmacovigilance required need not be the same for all loiasis/LF/onchocerciasis co-endemic, or potentially co-endemic, areas [38]. The following guidelines have thus been recommended for the initiation of PELFs in *L. loa *endemic areas where mass treatment with Mectizan for onchocerciasis is indicated:

• In those areas that have already received two or more cycles of mass treatment with Mectizan for onchocerciasis with good coverage (i.e. ≥ 65% therapeutic coverage of the total population or ≥ 80% therapeutic coverage of the eligible population), the level of *L. loa *microfilaremia in individuals is likely to be reduced far below levels associated with encephalopathy and other SAEs. Consequently, it can be recommended that the addition of albendazole could proceed with *enhanced passive surveillance* as currently recommended in the above-mentioned MEC/TCC guidelines for the treatment of onchocerciasis with Mectizan in areas co-endemic for loiasis [34].

• In areas that have received no previous mass treatment with Mectizan for onchocerciasis, one round of prior mass treatment, or have had poor prior mass treatment coverage, *active surveillance* similar to that employed at the initiation of the GPELF should be undertaken until a minimum of 15,000 individuals has been assessed [40]. This active surveillance should be undertaken only in those areas where all of the medical safety mechanisms for handling potential SAEs are well in place, as outlined in the MEC/TCC guidelines for treatment in *L. loa *endemic areas [34]. Based on the prior data from Cameroon, statistical considerations indicate that assessment of 15,000 treated individuals would permit the detection of any significant increase in SAEs that might be associated with the addition of albendazole to Mectizan in such *L. loa *endemic areas [41]. *If no increase is seen during this active surveillance, enhanced passive surveillance could then be instituted.*

It should be noted that this recommendation does not apply to areas where LF and loiasis endemicity overlap with areas where mass treatment with Mectizan for onchocerciasis is not indicated or where onchocerciasis is not endemic; alternative strategies for LF elimination will have to be considered in these areas.

### Outlook for 2006

#### Onchocerciasis

Among the new mass treatment programs with Mectizan for onchocerciasis to be launched in 2006 are one in the Rutshuru-Goma focus located in North Kivu Province, Democratic Republic of the Congo (DRC) and another in the Littoral I focus in Littoral Province, Cameroon, an area where the MEC/TCC guidelines for the treatment of onchocerciasis with Mectizan in areas co-endemic for onchocerciasis and loiasis will be implemented [34]. In 2006/2007, the MEC expects to receive new requests for Mectizan for endemic foci in Angola, DRC, and Uganda for the last remaining mass treatment programs with Mectizan for onchocerciasis to be launched in Africa. Once these programs are ongoing, all of the geographic areas eligible for mass treatment for onchocerciasis worldwide will be receiving Mectizan on an annual basis. Projections indicate that, by 2010, there will be over 100 million people receiving annual treatment with Mectizan for onchocerciasis in endemic countries, with more than 86 million of these being in the APOC region [personal communication L. Yameogo]. In light of this projection, consideration is being given to extending APOC operations beyond 2010 to assure sufficient time for all member countries to establish sustainable mass treatment programs with Mectizan for onchocerciasis [42].

#### Lymphatic Filariasis

In 2006, the PELFs in Burkina Faso and Ghana plan to join the ranks of those PELFs reaching 100% geographic coverage of districts where the co-administration of albendazole and Mectizan is indicated. Moreover, it is likely that a PELF in Sierra Leone will be initiated this year which is integrated with the existing onchocerciasis control program.

#### Integrated control programs

In several countries in Africa (e.g. Benin, Burkina Faso, Ghana, and Mali among others), national onchocerciasis control activities and national PELFs have been integrated since their intervention strategies are so similar. Other examples of integration are regions in Cameroon, DRC, Nigeria, and Uganda where Vitamin A is distributed during mass treatment with Mectizan for onchocerciasis [43,44]. In addition, in two states in Nigeria, MDA for onchocerciasis and schistosomiasis control are integrated with the LF elimination efforts. Uganda's PELF has recently been integrated into Child Days Plus which as part of its strategy includes deworming and immunization campaigns. It is expected that, in 2006 and onward, additional countries will initiate and intensify efforts to integrate onchocerciasis control with PELFs and with other disease control activities [45,46].

## Competing interests

MMA, NAYT-D, and BIT are employees of the Mectizan^® ^Donation Program which is funded by Merck & Co., Inc. and GlaxoSmithKline.

## Authors' contributions

All authors contributed equally to the preparation of this article.
